# Early clinical trial unit tumor board: a real-world experience in a national cancer network

**DOI:** 10.1007/s00432-023-05196-x

**Published:** 2023-07-25

**Authors:** L. Weiss, K. Dorman, M. Boukovala, F. Schwinghammer, P. Jordan, T. Fey, K. Hasselmann, M. Subklewe, V. Bücklein, R. Bargou, M. Goebeler, C. Sayehli, S. Spoerl, F. Lüke, D. Heudobler, R. Claus, I. von Luettichau, S. Lorenzen, S. Lange, C. B. Westphalen, M. von Bergwelt-Baildon, V. Heinemann, C. Gießen-Jung

**Affiliations:** 1 Department Medicine III (Hematology and Oncology), LMU University Hospital Munich, Munich, Germany; 2 Early Clinical Trials Unit, Department of Internal Medicine II, University Hospital Würzburg, Würzburg, Germany; 3 Comprehensive Cancer Center Mainfranken, University Hospital Würzburg, Würzburg, Germany; 4 Department of Medicine II (Gastroenterology), Klinikum Rechts Der Isar, Technical University of Munich, Munich, Germany; 5 Department of Pediatrics and Children’s Cancer Research Center, TUM School of Medicine, Kinderklinik München Schwabing, Technical University of Munich, Munich, Germany; 6 Department of Internal Medicine 5 (Hematology and Clinical Oncology), Friedrich-Alexander-Universität Erlangen-Nürnberg, University Hospital Erlangen, Erlangen, Germany; 7 Department of Internal Medicine III (Hematology and Oncology), University Hospital Regensburg, Regensburg, Germany; 8 Department of Hematology and Clinical Oncology, University Medical Center Augsburg, Augsburg, Germany; 9 Comprehensive Cancer Center (CCC Munich LMU), LMU University Hospital Munich, Munich, Germany; 10 Division of Personalized Tumor Therapy, Fraunhofer Institute for Toxicology and Experimental Medicine, Regensburg, Germany; 11 German Cancer Consortium (DKTK), Partner Site Munich, Munich, Germany; 12 Bavarian Cancer Research Center (BZKF), Munich, Germany

**Keywords:** Early clinical trial, ECTU, Clinical trials, Precision oncology, Interdisciplinary, Tumor board

## Abstract

**Purpose:**

Early clinical trials are the first step into clinical therapies for new drugs. Within the six Bavarian university-based hospitals (Augsburg, Erlangen, Regensburg, Munich (LMU and TU), Würzburg) we have enrolled a virtual network platform for patient discussion.

**Methods:**

The virtual Early Clinical Trial Unit Tumor Board (ECTU Tumor Board) is a secured web-based meeting to evaluate early clinical trial options for patients, where representatives from local ECTUs participate. We retrospectively analyzed patient cases discussed between November 2021 and November 2022.

**Results:**

From November 2021 to November 2022, a total of 43 patients were discussed in the ECTU Tumor Board. Median age at diagnosis was 44.6 years (range 10–76 years). The median number of previous lines of therapies was 3.7 (range 1–9 therapies) including systemic treatment, surgery, and radiation therapy. A total of 27 different tumor entities were presented and 83.7% (36/43) patients received at least one trial recommendation. In total, 21 different active or shortly recruiting clinical trials were recommended: ten antibody trials, four BiTE (bispecific T cell engager) trials, six CAR (chimeric antigen receptor) T-cell trials, and one chemotherapy trial. Only six trials (28.6%) were recommended on the basis of the previously performed comprehensive genetic profiling (CGP).

**Conclusion:**

The ECTU Tumor Board is a feasible and successful network, highlighting the force of virtual patient discussions for improving patient care as well as trial recruitment in advanced diseases. It can provide further treatment options after local MTB presentation, aiming to close the gap to access clinical trials.

## Introduction

Early clinical trials are a fundamental step from preclinical drug development into clinical practice. A wide range of opportunities opened up by cancer genomics and the promise of personalized medicine shifting therapeutic strategies from tumor entity-based to tumor-agnostic and molecularly driven (gene-directed) therapy (Tsimberidou et al. 2020). Gene sequencing has revolutionized the process of identifying novel molecular targets for drug discovery and the analysis of new biomarkers in clinical trials offers a promising way forward to achieve an efficient transition into new drug development (Debouck and Metcalf 2000). In the last years the field of gene and cell therapy has been developing rapidly, following the first approved therapies which have shown life-changing effects for the patients (Bulaklak and Gersbach 2020). This new era of cancer treatment ends the “one chemo fits all” approach, by aiming at tailoring an effective and safe targeted therapy in all lines of systemic therapy leading to a variety of molecularly guided clinical trials.

Early clinical trial units (ECTU) are specialized clinical trial units for experimental tumor therapies, which are sufficiently equipped and staffed to conduct phase I, phase I/II, and phase II trials with new therapeutic approaches. Cancer patients get access to new innovative therapies in a setting led by highly experienced physicians, and operated in accordance with ICH GCP (International conference on harmonisation—Good clinical practice). Phase I/II trials address mostly patients with advanced disease that are unresponsive or face progressive disease despite standard therapies, although early clinical trials for first-line therapies exist as well. Molecularly targeted agents often differ from standard cytotoxic agents by their administration schedules and routes, their toxicity profiles and/or the assessment of their antitumor activity (Le Tourneau et al. 2010). In order to accelerate early translation of novel therapies for patients with hematological and oncological diseases, early clinical trials are directly linked to innovative preclinical research.

The state of Bavaria, located in the south-east of Germany, is the largest German state by area and with over 13 million inhabitants. A total of six university hospitals are located in the Bavarian state: university hospital Augsburg, university hospital Erlangen, Munich (LMU Klinikum and Klinikum rechts der Isar), university hospital Regensburg, and university hospital Würzburg. The university hospitals or academic centers, each with a local designated ECTU, and the associated Comprehensive Cancer Centers (CCC), are connected through the Bavarian Cancer Research Center (BZKF; https://bzkf.de/). By joining forces and close networking, cancer research can be optimized and accelerated. Furthermore, specialized and disease-focused preclinical and clinical research infrastructures and ideas can be exchanged, and access to clinical trials is shared. The multi-sited workgroup for early clinical trials was founded 2020 with the aim to optimize access to innovative treatment options for cancer patients in Bavaria by connecting the Early Clinical Trial Units of all six Bavarian university hospitals. The ECTU network also includes the Bavarian Phase I Network in Pediatric Oncology (KIONET; https://www.kionet-bayern.de/).

Due to the complexity of early clinical trials with the need for specialized facilities and experienced and trained staff, the number of early clinical trials recruiting at each university hospital is more limited compared to phase III trials. In order to increase access to innovative treatment options, particularly early clinical trials, for cancer patients beyond their hometown even in underserved regions, the ECTU Tumor Board was founded.

Interdisciplinary patient discussions and disease-specific Tumor Boards are a very well-established tool in healthcare to address important challenges such as interdisciplinary collaboration (Lamb et al. 2012; Soukup et al. 2016). Unlike conventional Tumor Boards, which are usually held in a single hospital or institution, the ECTU Tumor Board connects all six university hospitals in Bavaria. Therefore, regulations regarding data-privacy and patient-registration had to be fulfilled rigorously.

Here we describe the development and implementation of a newly founded Early Clinical Trial Unit Tumor Board (ECTU Tumor Board) which is hosted virtually within the Bavarian university-based hospitals.

## Materials and methods

We performed an exploratory analysis of the patients presented in the newly founded ECTU Tumor Board. The acquisition of primary data took place between November 2021 and November 2022 via the ECTU patient platform.

### Workflow

Oncological patients are presented in one of the local organ-/disease-specific Tumor Boards for example hematological-, gastrointestinal-, gynecological-, urological Tumor Board, at time of initial diagnosis. In accordance with the guidelines and individual patient characteristics the Tumor Board can help to identify a suitable time point for comprehensive genomic profiling (CGP) (Mosele et al. 2020). Depending on the tumor entity, the time point of extended molecular testing can differ drastically. CGP results are then discussed in the local Molecular Tumor Board. In an advanced disease or progressive disease despite standard therapies, including also the therapy recommended by the local Molecular Tumor Board, early clinical trials can be evaluated for medically fit patients. If the patient does not qualify for local trials or no clinical trial is suitable, a screening via the ECTU Tumor Board can be evaluated. Before the ECTU Tumor Board registration, patients need to be asked whether they would be willing to travel to another site for a clinical trial. General inclusion criteria regarding the Tumor Board are age below 75 years and an ECOG performance status of 0–1. We also encourage hematological patients with high-risk-disease during or after indicated standard therapies to be discussed in the ECTU Tumor Board (Table 1).

**Table 1:** Tumor entities presented in the ECTU tumor board

Diagnosis	Number of patients
Colorectal cancer	7
Pancreatic cancer	5
Non-small-cell lung cancer	2
Rhabdomyosarcoma	2
Medullary thyroid cancer	3
Renal cancer	2
Adrenocortical carcinoma	2
Vaginal cancer	1
Diffuse large B-cell lymphoma	1
Non-Hodgkin lymphoma	1
Cervical cancer	1
Acute myeloid leukemia	1
Osteosarcoma	1
Oesophagogastric junctional carcinoma	1
Anal cancer	1
Appendix cancer	1
Bile duct cancer	1
Breast cancer	1
Urothelial carcinoma	1
Parotis cancer	1
Meningioma	1
Angiosarcoma	1
Ewing-Sarcoma	1
Leiomyosarcoma	1
Synovialsarcoma	1
CUP	1
NEC	1
**Total**	**43**

Since its implementation in November 2021, the access to the Tumor Board has been limited to physicians of the six Bavarian university hospitals. Patient registration, considering data-privacy, was strictly performed via the ECTU platform. The Tumor Board was held virtually biweekly, and was then switched to a monthly virtual meeting. The participants consisted of the ECTU members of the six Bavarian sites, and representatives of each local MTB. Each patient was discussed and was evaluated for ongoing and soon to be initiated trials at all sites, therefore, the representative of each site could inform about the current status of the site’s clinical trials. After discussion, a Tumor Board recommendation was uploaded to the ECTU platform and can be downloaded by the treating physician anytime.

### ECTU platform

To ensure the regulations regarding data-privacy and patient-registration are followed, we developed a new web-based ECTU patient platform consisting of a secure website with a data privacy concept authorized by the data security manager of the LMU university hospital in Munich. Access to this platform is strictly regulated to representatives of the Bavarian ECTU and MTB sites. Besides the year of birth, the process of patient registration requires no personally identifiable information. Patient registration, download of registered patients and upload of Tumor Board recommendations can be performed only via platform access. The platform was programmed to down- and upload all recommendations automatically. The access to the ECTU Tumor Board recommendations is only permitted to the site who had registered the patient.

### Patients

A clinical database was established including the following information for each patient: year of birth, age at diagnosis, date of initial diagnosis (month and year), tumor entity, tumor histology, CGP results, previous therapies, other malignancies or relevant comorbidities, elevated hepatic laboratory results, and Tumor Board recommendation.

### Statistical analysis

All statistical analyses were performed using SPSS version 29 for Windows (SPSS Inc, Chicago, IL).

## Results

**Fig. 1:**
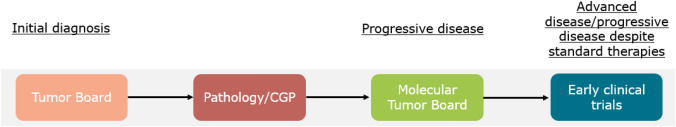
Patient algorithm for early clinical trials

Within the first year of the ECTU Tumor Board, starting on 26th November 2021, a total of 12 Tumor Boards were held with on average of 11 participants from four to five sites. 46 patients were registered via our ECTU platform, however, only 43 were discussed in the Tumor Board, since three patients were withdrawn due to worsening of general condition or death (Fig. 1).

In our cohort, median age at diagnosis was 44.6 years (range 10–76 years). Since the KIONET-network is included in our ECTU Tumor Board, four pediatric patients were presented with a median age of 15 years (range 10–17 years). Median age for the adult patients (≥ 18 years) was 51.4 years (range 19–76 years). The median number of previous lines of therapies was 3.7 (range 1–9 therapies) including systemic treatment, surgery, and radiation therapy.

**Fig. 2:**
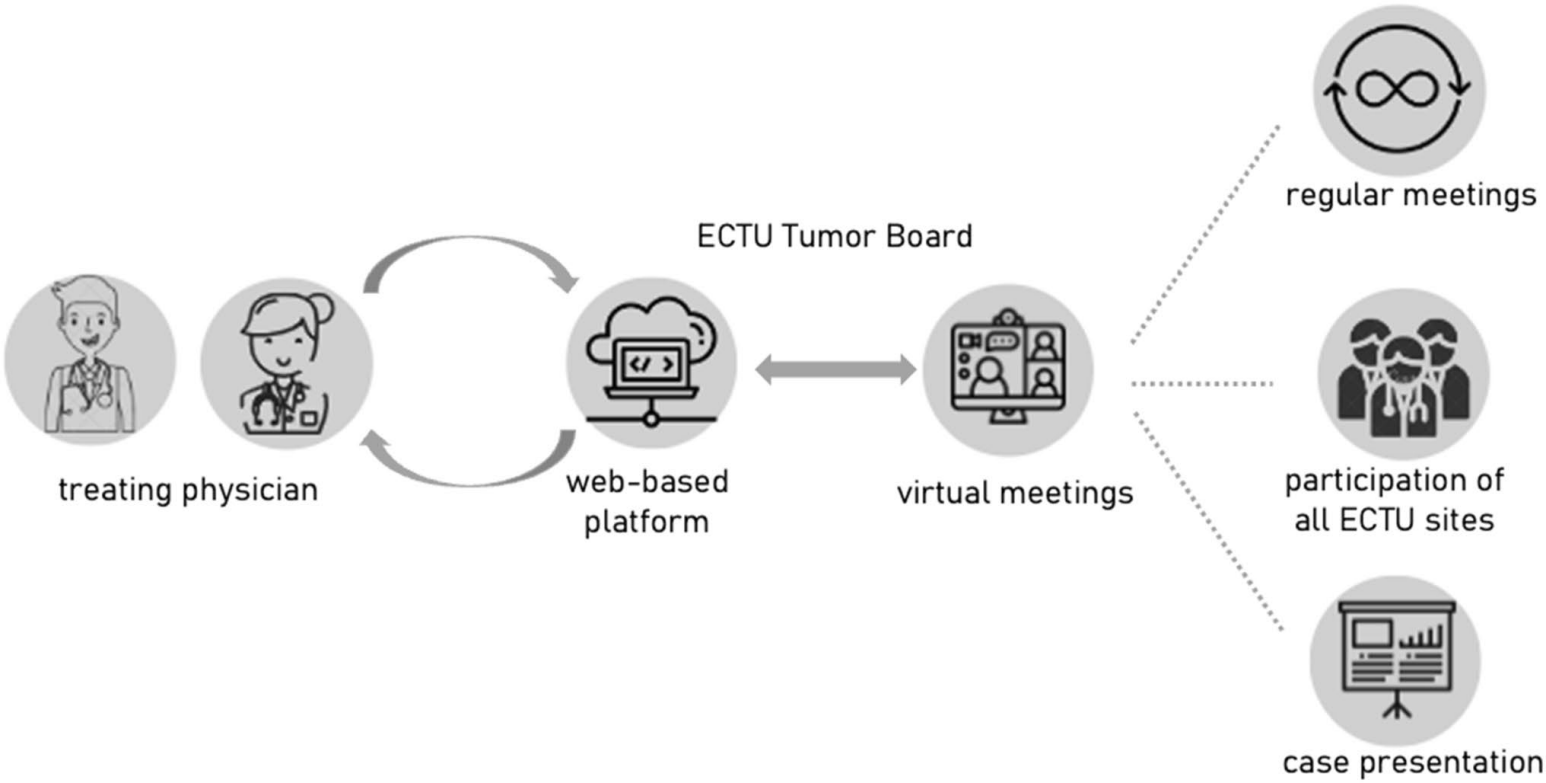
Structure of the ECTU tumor board

A total of 27 different tumor entities (Fig. 2), ranging from solid tumors of the gastrointestinal tract, gynecological tumors, as well as head and neck cancers, to hematological neoplasms or sarcomas, were presented to the ECTU Tumor Board. The most common diagnosis was colorectal cancer (*n* = 7), followed by pancreatic cancer (*n* = 2). All patients suffered from metastatic disease. On average, 19.1 months after initial diagnosis the patients were presented to the ECTU Tumor Board.

A total of 36 patients (83.7%) received at least one ECTU Tumor Board recommendation. Nine patients received recommendations for two or more different clinical trials, three patients were recommended to apply for an early-access program. In total, 21 different ongoing or shortly recruiting clinical trials were recommended: ten antibody trials, four BiTE (Bispecific T-Cell Engager) trials, six CAR (chimeric antigen receptor) T cell trials, and one chemotherapy trial.

Out of the 21 recommended trials, only six trials (28.6%) were recommended on the basis of the previously performed CGP, since their inclusion criteria were based on molecular alterations that are covered in common panels. Nine (42.9%) trials had a specific screening part for biomarkers that are not covered by common CGP panels, five (23.8%) trials were biomarker-independent and only included patients based on certain tumor entities and line of therapy, and four (19.0%) trials used a screening for a non-molecular marker (HPV, HLA-type).

A total of 35 patients (81.4%) were presented to the Tumor Board with previously performed CGP where across all patients 47 different alterations were found. The most common alterations were KRAS non-G12C (*n* = 11), followed by TP53 (*n* = 9), APC (*n* = 5), SMAD4 (*n* = 4), TMB intermediate (*n* = 4), and PIK3CA (*n* = 3). For further information please refer to Table 2.

**Table 2:** Molecular alterations and CGP characteristics of discussed patients

Alteration	Number of patients
KRAS (non G12C)	11
TP53	9
APC	5
SMAD4	4
TMB intermediate	4
PIK3CA	4
RET	3
BRAF (non V600E)	2
MYC	2
FBXW7	2
PAX7-FKHR (FOXO1)	2
PTEN	2
ARID1A	1
ATM	1
AXIN2	1
BAP1	1
CHEK2	1
CHEK2	1
CK4	1
CTNNB1	1
DNMT1	1
ERBB2	1
ETNNB1	1
FANCD2	1
FGFR1	1
FGFR2	1
FGFR3	1
FGFR4	1
GLI1	1
LATS1	1
MAD1L1	1
MAG	1
MAP2K1	1
MITF	1
NRAS	1
PBRM1	1
RB1	1
RECQL4	1
RIT1	1
SHH	1
SYT	1
TCF7L2	1
TERT	1
TMB high	1
VHL	1
XPO1	1
ZFHX3	1

## Discussion

The Bavaria-wide ECTU Tumor Board is the first step for a Bavaria-wide Tumor Board, highlighting the force of network structures for both improving patient care and trial recruitment in advanced diseases.

To connect multiple sites, digital technologies can be used to support healthcare provision in many ways by enabling access to medical care for hard-to-reach populations or facilitating patient care for physicians without increasing the workload (Kaiser et al. 2021; Webster 2020). The COVID-19 pandemic has had significant impact on the rapid adoption of digital tools and technologies for the treatment of patients (Bokolo Anthony 2020). Further sites can be connected easily, and participate in a well-adapted virtual Tumor Board.

Patients with various kinds of advanced malignancies that are either relapsed or refractory to standard therapy or for which no curative therapy exists are ideal candidates for early clinical trials (Ivy et al. 2010). The patient cohort of the Bavarian ECTU Tumor Board (low median age medically fit, progressive disease despite standard therapy) is the first selection-step toward early clinical trial recruitment. The main reason for cancer patients not entering a trial is the lack of an available and recruiting trial and failure to meet inclusion criteria (Corrie et al. 2003). The preselection of suitable patients starts with the general inclusion criteria (medically fit, aged < 75 years). Further selection occurs by discussion in the ECTU Tumor Board with representatives of all ECTUs. Thereby, the rate of screening failure can be reduced. Besides previous therapies, we ask for further important information for trial screening in the process of patient registration such as elevated hepatic laboratory values, relevant comorbidities or other concurrent malignancies. The major difference to a common study-register is particularly evident in the individual discussion of each patient: within the ECTU Tumor Board the treating physician and the ECTU investigators meet virtually. Major in- and exclusion criteria can be discussed and more importantly each representative of the ECTUs can give an exact update on the current recruitment status of all active or soon to be recruiting trials. Knowledge of trials or cohorts that are on hold or fully recruited can be communicated on a highly current basis, such as open cohorts or waiting time for screening and slot request.

Mediating clinical trials beyond patients’ hometowns within the Bavarian state can increase the chances to be successfully screened and enrolled in clinical trials for late-stage cancer patients. Only 3–5% of cancer patients participate in clinical trials although an US-based interview indicated over 30% of adults would be willing to participate in a clinical cancer trial if only asked (Comis et al. 2003). Therefore, other factors seem to impede participation such as distance to a study center and the lack of awareness about suitable clinical trials (Baquet et al. 2006; Kadam et al. 2016).

With 83.7% of the patients receiving a Tumor Board recommendation we are able to show that even in the implementation phase, a multi-site patient discussion serves as a powerful and successful tool for increasing treatment options for patients and trial recruitment for ECTUs. The distribution of therapy modalities with the highest proportion representing antibody trials, followed by cellular therapies with BiTEs and CAR T cells reflects the success of genetic medicine (Bulaklak & Gersbach 2020) and a trend toward immune-oncology and cellular therapies.

Heinrich et al. described the first consecutive 1000 patients presented in the CCCMunich^LMU^ Molecular Tumor Board, where 801 patients underwent successful CGP. No alteration was found in 18.4% (*n* = 168), and 257 (28.1%) did not receive a MTB recommendation due to missing a targetable mutation although at least one alteration had been identified (Heinrich et al. 2022). In total, 532 patients who underwent CGP did not receive a recommendation for therapy (58.2%). These results are in line with current data. Trédan and coworkers descripted the ProfiLER trial, where 2579 adult and pediatric patients with solid and hematological cancer were enrolled and 1980 patients (76.8%) underwent molecular profiling and consecutive presentation to a Molecular Tumor Board. While 948 patients had no actionable alteration, 333 did not receive a recommendation although at least one alteration was found (Trédan et al. 2019). Despite CGP, 64.7% (1281/1980) did not receive a recommendation. Other published data range the rate of percentage of patients not receiving a therapeutic recommendation after CGP between 38 and 61.9% (Gambardella et al. 2021; Kato et al. 2020; Larson et al. 2021).

In our cohort, we analyzed the clinical trials recommended in the ECTU Tumor Board based on the main criteria of inclusion. Only six out of 21 trials (28.6%) were dependent on previous screening with common CGP panels. The majority of recommended trials uses screening tools independently of previous CGP.

Since initially we expected a high rate of molecularly based trials, we strongly recommended the ECTU Tumor Board only for patients who had been initially presented to a local MTB. The importance of a Molecular Tumor Board remains unchanged and the benefit for patients with actionable alterations remains inviolable. However, the rate of therapeutic recommendations based on actionable targets is low. Clinical trials, mainly early phase trials, are therefore a feasible alternative for access to innovative treatment also for patients without actionable alterations.

**Fig. 3:**
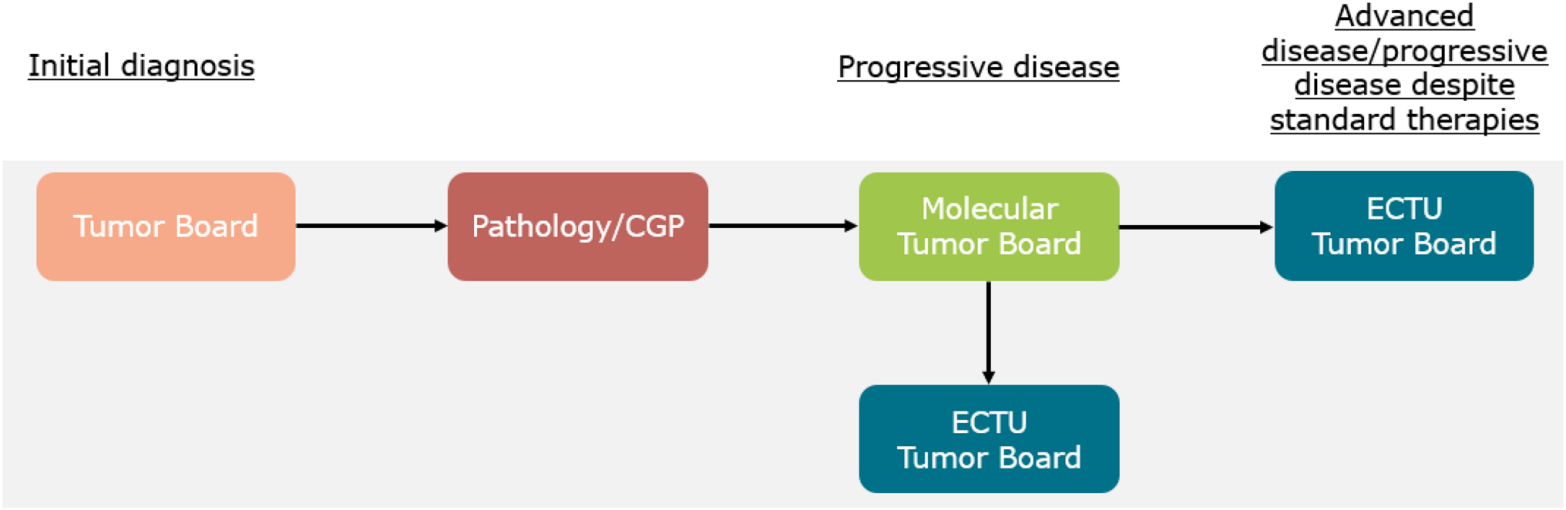
New patient algorithm for the ECTU tumor board

Based on these results, we adapted our algorithm for patient registration (Fig. 3): patients who were presented to a local molecular Tumor Board and did not receive a therapeutic recommendation, either due to unsuccessful testing without the intention of re-testing, or the absence of actionable alterations, should be allocated to the ECTU Tumor Board immediately. For patients with actionable alterations, implementation of MTB recommendation should be prioritized. After tumor progression, an ECTU Tumor Board presentation should be evaluated if applicable (Fig. 3).

In contrast to the MTB which can be held locally, the benefit of the ECTU Tumor Board shows only by connecting multiple sites. By sharing local portfolios of ECTUs the network can offer a wider range of different innovative clinical trials. The possible benefits of the ECTU Tumor Board include higher trial recruitment, especially for slowly and difficult-to-recruit trials, and a larger catchment area. The cooperation within the ECTU Tumor Board resulted in a close network structure in die field of early clinical trials. We are convinced that the advantage will increase if other ECTU sites, not limited to Bavaria. Our goal is therefore to gradually add further sites.

Bavarian-wide access to diagnostic platforms allows for joint screening of a large patient population and facilitates the implementation of a broad biomarker driven trial program. Connecting local ECTUs to one collaborative work-group increases the recruitment potential by recommending innovative clinical trials beyond the patients’ hometown and also improving access for patients in remote areas. Beyond patient presentation, the ECTU Tumor Board opens opportunities by connecting ECTU-sites. By forming a network between the six Bavarian university hospitals and their ECTUs, with a catchment area of 13 million inhabitants, the network connects existing ECTUs and promises further potential. Currently, patient follow up for discussed patients with focus on inclusion in an early clinical trial as well as outcomes is in progress.

## Data Availability

Available from the first author upon reasonable request.
